# Green synthesis, characterization and applications of *Phyllanthus emblica* fruit extract mediated chromium oxide nanoparticles

**DOI:** 10.1186/s11671-024-04006-8

**Published:** 2024-04-16

**Authors:** Easha Fatima, Iqra Arooj, Mehvish Javeed, Jian Yin

**Affiliations:** 1 Department of Microbiology and Molecular Genetics, Faculty of Life Sciences, The Women University, Multan, 66000 Pakistan; 2 CAS Key Lab of Bio-Medical Diagnostics, Suzhou Institute of Biomedical Engineering and Technology, Chinese Academy of Sciences, Suzhou, 215163 Jiangsu China

**Keywords:** Chromium oxide, Nanoparticles, *Phyllanthus emblica*, Characterization, Antimicrobial

## Abstract

The green synthesis of metallic nanoparticles is attributable towards diverse applications in various fields, recently. In this research, we report simple and eco-friendly synthesis of chromium oxide (Cr_2_O_3_) nanoparticles using the fruit extract of *Phyllanthus emblica* as a reducing and capping agent. The absorbance peaks at 350 nm and 450 nm validated the nanoparticle formation in UV–visible spectrum. FTIR spectrum revealed the nature of functional groups. The crystalline properties of nanoparticles were ascertained by XRD analysis. EDX spectrum corroborated the elemental composition of nanoparticles in which chromium and oxygen constituted 68% of total weight. SEM images demonstrated agglomeration of nanoparticles resulting in the formation of large irregularly shaped flakes. Cr_2_O_3_ nanoparticles demonstrated excellent antimicrobial properties against 11 bacterial isolates and 1 fungal isolate. The largest inhibition zone (53 mm) was measured against *A. baumannii* while the smallest inhibition zone (26 mm) was recorded against *S. aureus*. Minimum inhibitory concentration (MIC) values were < 1 µg/ml for all microbes. However, the synthesized nanoparticles did not reveal synergism with any of the selected antibiotics (FICI values > 1). Nanoparticles possessed potent anti-biofilm powers with maximum (77%) inhibition of *E. coli* biofilms and minimum (45%) inhibition of *S. enterica* biofilms. Photocatalytic activity of Cr_2_O_3_ nanoparticles was evaluated to determine their efficacy in environmental bioremediation. Outcomes demonstrated degradation of methyl red (84%) but not of methylene blue dye. Furthermore, the Cr_2_O_3_ nanoparticles displayed considerable antioxidant (43%) as well as anti-inflammatory (44%) potentials. Hence, the present study accounts for the versatile applications of *P. emblica*-mediated Cr_2_O_3_ nanoparticles which could be pursued for future biomedical and environmental applications.

**Graphical Abstract:**

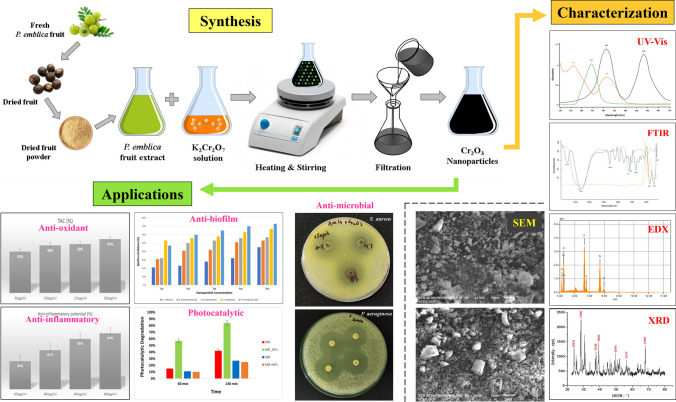

## Introduction

Nanotechnology has gained significant attention in recent years due to the unique characteristics and widespread applications of nanoparticles across various fields including, but not limited to, medicine, energy and electronics [1]. The distinctive characteristics of nanoparticles, including their diminutive size (typically ranging from 1 to 100 nm), expansive surface area and adjustable surface chemistry, render them perfect for precise drug delivery, improved antimicrobial efficacy and environmental remediation [2].

Scientists have utilized a range of metals such as gold, silver, copper, zinc, iron, chromium, platinum and palladium to synthesize nanoparticles, enabling diversified applications [3]. The nanoparticle synthesis methods encompass physical, chemical and biological approaches [4]. Among the biological methods, one notable subcategory is the utilization of plant extracts as both reducing and capping agents, commonly referred to as green synthesis. Green synthesis has been increasingly recognized as a sustainable and environment friendly method in recent years [5]. Numerous plant extracts have been successfully employed in this process [6]. Plant extracts are rich in various phytochemicals, such as phenols, flavonoids and terpenoids, which inherently possess reducing and stabilizing capabilities [7]. These substances can efficiently transform metal salts into nanoparticles under gentle reaction conditions, thereby eliminating the requirement for harmful chemicals and high-energy procedures. Nanoparticles produced in this way have a well-defined size distribution and excellent stability [8]. Given its sustainable methodology and broad potential for various applications, the green synthesis of nanoparticles using plant extracts offers immense potential in the realm of nanotechnology applications [9].

Antibiotic resistance, exacerbated by inappropriate antibiotic use and bacterial genetic modifications, is a major global health issue which has increased morbidity and mortality, thereby escalating healthcare costs [10–12]. Addressing this problem necessitates a comprehensive approach including, responsible antibiotic usage, development of new drugs and exploration of innovative treatments. Nanoparticles have emerged as a potential solution to combat antibiotic-resistant bacteria [13]. Scientists have managed to bypass bacterial resistance by coupling antibiotics with nanoparticles, enabling successful treatment even amidst resistance [14]. Moreover, nanoparticles can be designed to selectively attack specific bacteria, leaving healthy cells unharmed, thus reducing cytotoxicity and other adverse effects [15]. Their ability to infiltrate biofilms, the protective shields created by bacteria, further amplifies their effectiveness against resistant microbes [16]. Although further studies are required to fully comprehend the mechanisms and fine-tune nanoparticle-based therapies, these developments provide a ray of hope in the struggle against antibiotic-resistant bacteria and the pressing demand for novel therapeutic options.

*Phyllanthus emblica,* commonly known as Indian gooseberry, is a fruit-bearing plant that has been utilized in traditional medicine for centuries. Scientific studies have extensively investigated the potential medicinal benefits of this plant, revealing promising therapeutic properties [17]. Furthermore, various metallic nanoparticles, including copper, silver, gold, magnesium oxide, iron oxide and zinc sulfide nanoparticles, have been successfully synthesized using extracts derived from *P. emblica* plant [18–23]. To the best of our knowledge, chromium oxide nanoparticles have not yet been synthesized using *P. emblica* fruit extract. So, we used green synthesis approach for synthesizing chromium oxide nanoparticles using *P. emblica* fruit extract as a stabilizing agent. We characterized these nanoparticles using various techniques and investigated their antimicrobial, anti-inflammatory, antioxidant and photocatalytic dye degradation potentials. It was anticipated that the nanoparticles created in this manner could prove to be a simple, quick, cost-effective and environmentally safe alternative to the antimicrobial drugs currently in use.

## Results

### Phytochemical and GC–MS analysis of P. emblica fruit extract

**Fig. 1:**
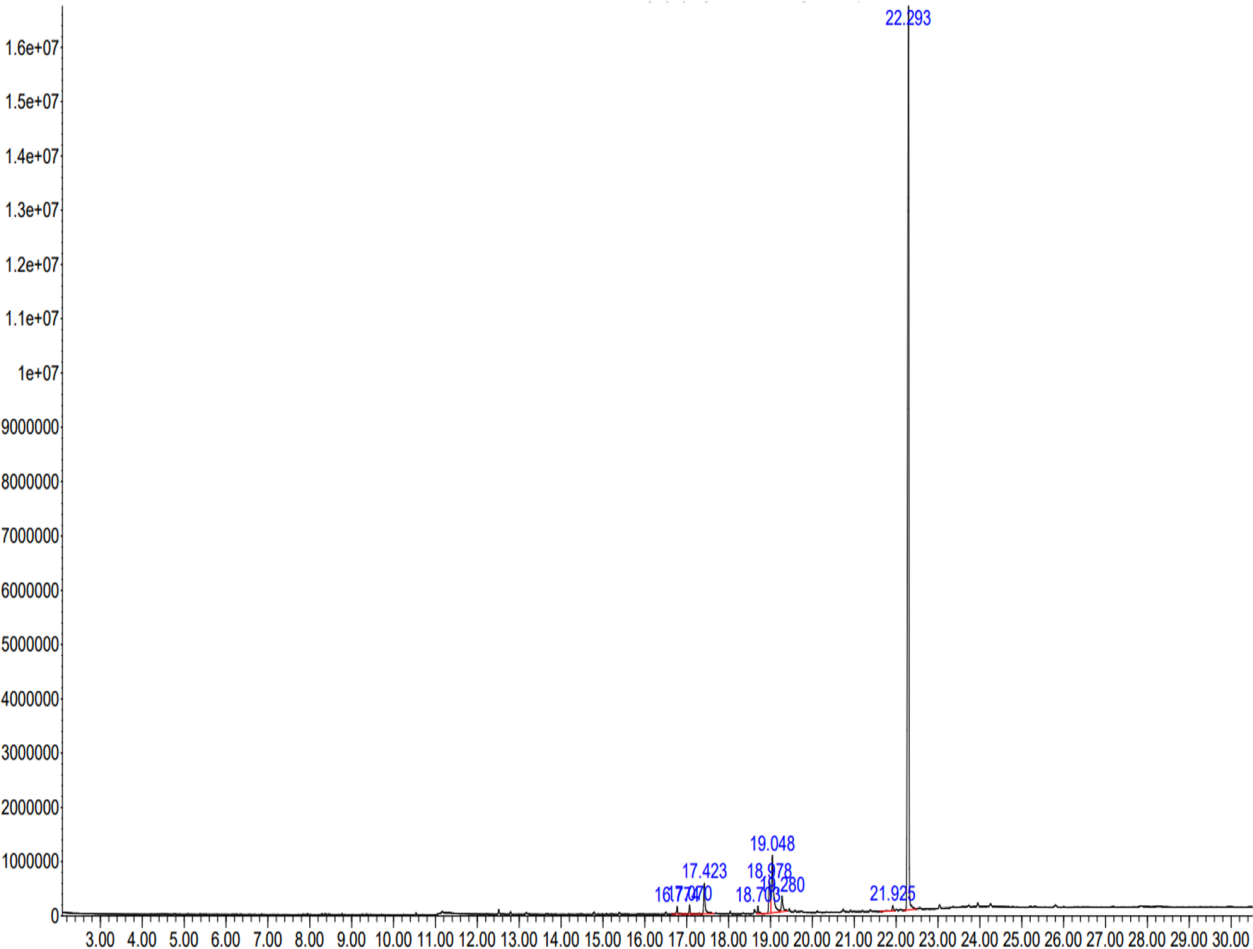
GC–MS chromatogram of *Phyllanthus emblica* fruit extract with retention time on X-axis and abundance on Y-axis

Qualitative phytochemical analysis of aqueous *P. emblica* fruit extract confirmed the presence of secondary metabolites such as carbohydrates, alkaloids, flavonoids, steroids, glycosides, phenols and tannins. Nonetheless, anthocyanins were not present. GC–MS analysis of the fruit extract was performed to identify the bioactive substances, which are responsible for the capping and stabilization of nanoparticles. The identified phytochemical compounds with their molecular formula, molecular weight, retention time and concentration (peak area percentage) are presented in Table 1 and Fig. 1, which signify the presence of nine bioactive phytochemicals in the extract.

**Table 1:** GC–MS spectral analysis of fruit extract of *Phyllanthus emblica*

No.	Retention time (RT)	Identified compound	Molecular formula	Molecular weight (g/mol)	% Peak area
1	16.774	7,9-Di-tert-butyl-1-oxaspiro(4,5)deca-6,9-diene-2,8-dione	C_17_H_24_O_3_	276.4	0.68
2	17.070	Hexadecanoic acid, methyl ester	C_17_H_34_O_2_	270.5	0.64
3	17.423	n-Hexadecanoic acid	C_16_H_32_O_2_	256.4	3.23
4	18.703	9-Octadecenoic acid, methyl ester	C_19_H_36_O	296.5	0.68
5	18.978	9,12-Octadecadienoic acid	C_18_H_32_O	280.4	3.22
6	19.048	Oleic acid	C_18_H_34_O_2_	282.5	7.77
7	19.280	Octadecanoic acid	C_18_H_36_O	284.5	1.82
8	21.925	Phthalic acid, di(1-tert-butoxyprop-2-yl) ester	C_17_H_24_O_5_	308.4	0.49
9	22.293	Bis (2-ethylhexyl) phthalate	C_24_H_38_O_4_	390.6	81.48

### Visual confirmation and UV–Visible spectroscopy of nanoparticles

**Fig. 2:**
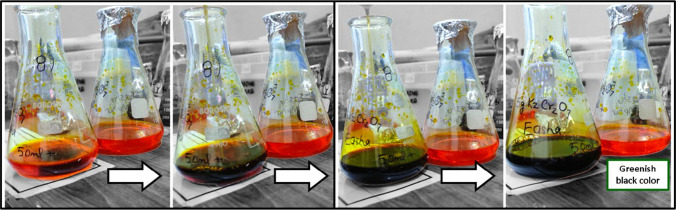
Bright orange colored potassium dichromate solution changing color to greenish-black upon formation of Cr_2_O_3_ nanoparticles

**Fig. 3:**
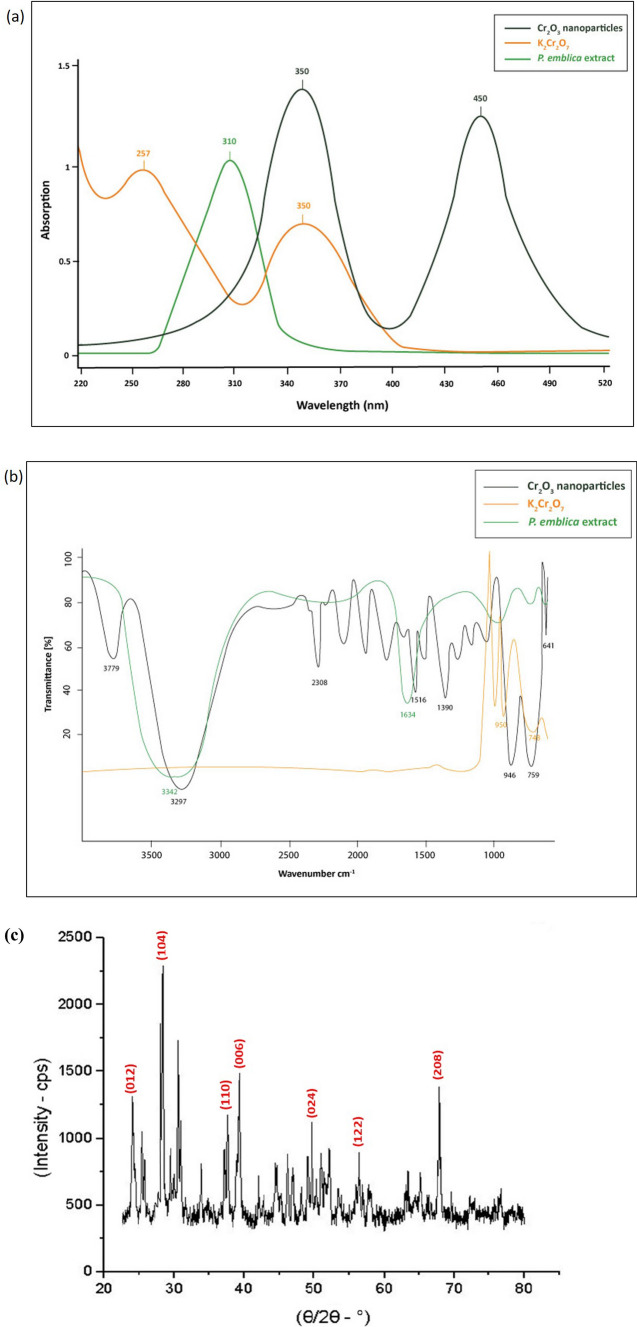

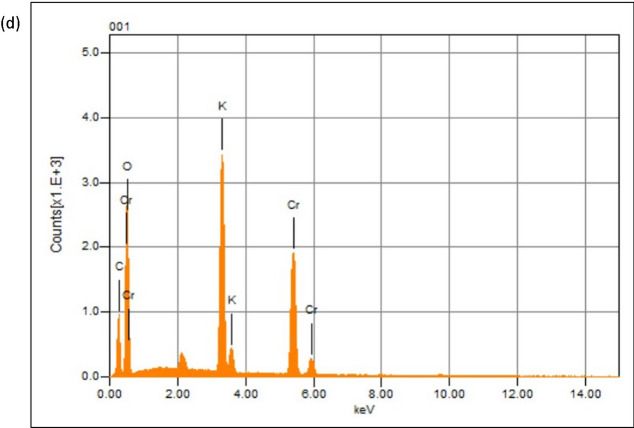
**a** UV–visible spectrum of *P. emblica* extract, potassium dichromate solution and Cr_2_O_3_ nanoparticles showing distinctive peaks. **b** FTIR spectrum of *P. emblica* extract, potassium dichromate solution and Cr_2_O_3_ nanoparticles. **c** XRD graph of Cr_2_O_3_ nanoparticles showing multiple peaks. **d** EDX graph of Cr_2_O_3_ nanoparticles showing clear Cr and O peaks

Upon mixing the potassium dichromate solution with *P. emblica* fruit extract, a greenish-black color appeared which served as a visual indication for the formation of Cr_2_O_3_ nanoparticles (Fig. 2). UV–visible spectroscopy peaks were recorded between 300 and 500 nm. Sharp peaks appeared at 350 nm and 450 nm, as shown in Fig. 3a. The peak at 350 nm, possibly, indicates the presence of precursor salt in the final solution while the peak at 450 nm is ascribed to the d-d transition of the metal during nanoparticles synthesis. Both peaks lie within previously reported wavelength ranges confirming the formation of nanoparticles. For potassium dichromate solution, two peaks appeared at 257 nm and 350 nm while for *P. emblica* extract, a single sharp peak was observed at 310 nm.

### Characterization of nanoparticles

**Fig. 4:**
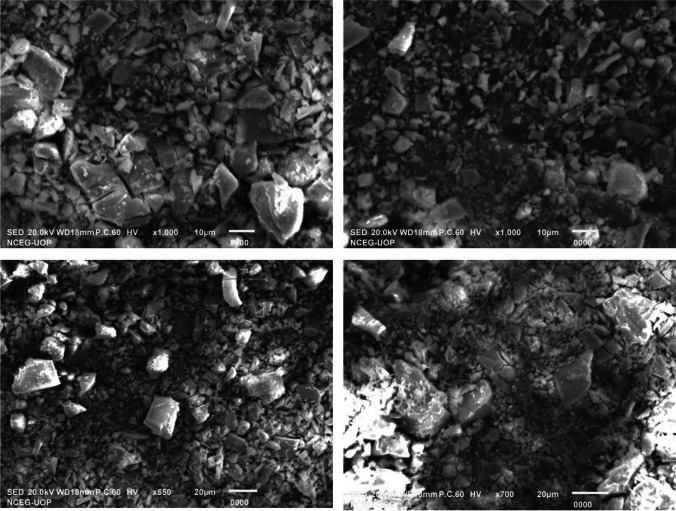
SEM images of Cr_2_O_3_ nanoparticles at different magnifications

FTIR spectrum of Cr_2_O_3_ nanoparticles revealed the presence of various functional groups. Peaks at 3779 cm^−1^ and 3297 cm^−1^ corresponded to O–H groups. Other peaks recorded in the spectrum were 2308 cm^−1^ (C≡N group), 1516 cm^−1^ (C=C group) and 1390 cm^−1^ (C–H bending). The FTIR signals at 946 cm^−1^, 759 cm^−1^ and 641 cm^−1^ pointed towards the Cr–O bond, which validated the formation of Cr_2_O_3_ nanoparticles (Fig. 3b). These results suggested that many bioactive phyto-molecules might be present on the surface of nanoparticles which were responsible for the formation of multiple peaks at different wavenumbers. FTIR spectrum of *P. emblica* extract revealed peaks at 3342 cm^−1^ (O–H group) and 1634 cm^−1^ (N–H bond), while that of potassium dichromate revealed peaks at 950 cm^−1^ and 748 cm^−1^, indicating metal oxide bonds. XRD spectrum of Cr_2_O_3_ nanoparticles presented peaks at diffraction angles of 24.6, 33.8, 37.5, 39.3, 49.6, 56.4, 69.5 and 76.2 which corresponded to the miller index planes of (0 1 2), (1 0 4), (1 1 0), (0 0 6), (0 2 4), (1 2 2), (2 0 8) and (2 2 0), according to the Joint Committee on Powder Diffraction Standards (JCPDS) (Fig. 3c). The appearance of sharp peaks indicated that the synthesized nanoparticles were crystalline in nature. EDX analysis distinctly showed the elemental composition of Cr_2_O_3_ nanoparticles. The presence of different elements was revealed in the form of clearly visible peaks. EDX graph manifested the presence of chromium and oxygen in the sample as Cr and O peaks, located between 0.0 and 0.6 keV (Fig. 3d). Potassium and some other elements were also present in the sample. Figure 4 presents the images obtained by SEM analysis which revealed that majority of the nanoparticles were present in the form of large agglomerates, which were not homogeneously distributed and were present as irregularly shaped flakes. Many of these agglomerates were very large and their sizes were even in the micrometer range.

### Antimicrobial activity and MIC

**Fig. 5:**
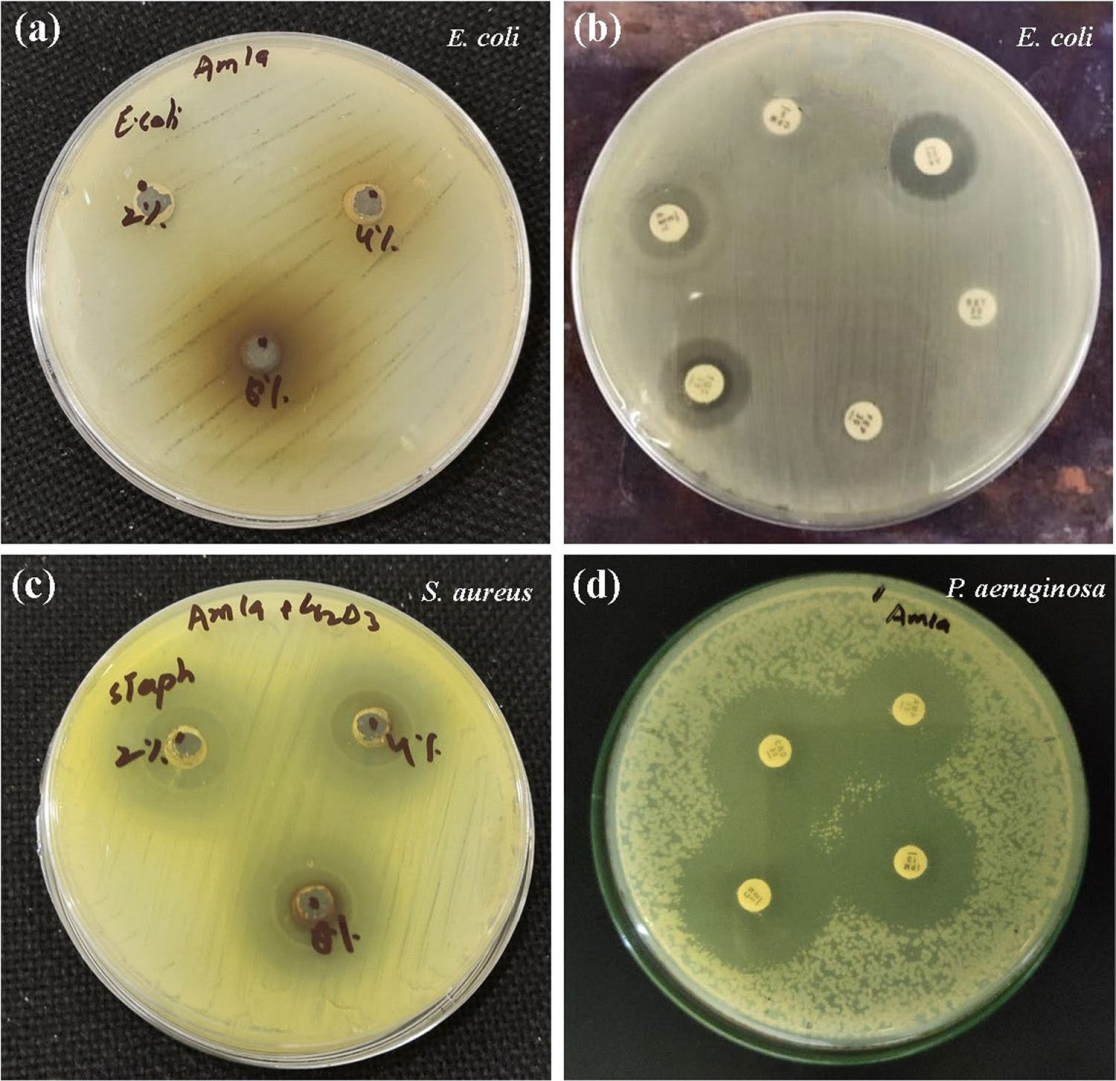
Agar plates showing **a** antibacterial activity of *P. emblica* extract, **b** antibiotic sensitivity testing of bacterial isolates, **c** antibacterial activity of Cr_2_O_3_ nanoparticles, **d** antibacterial activity of antibiotics conjugated with Cr_2_O_3_ nanoparticles

**Fig. 6:**
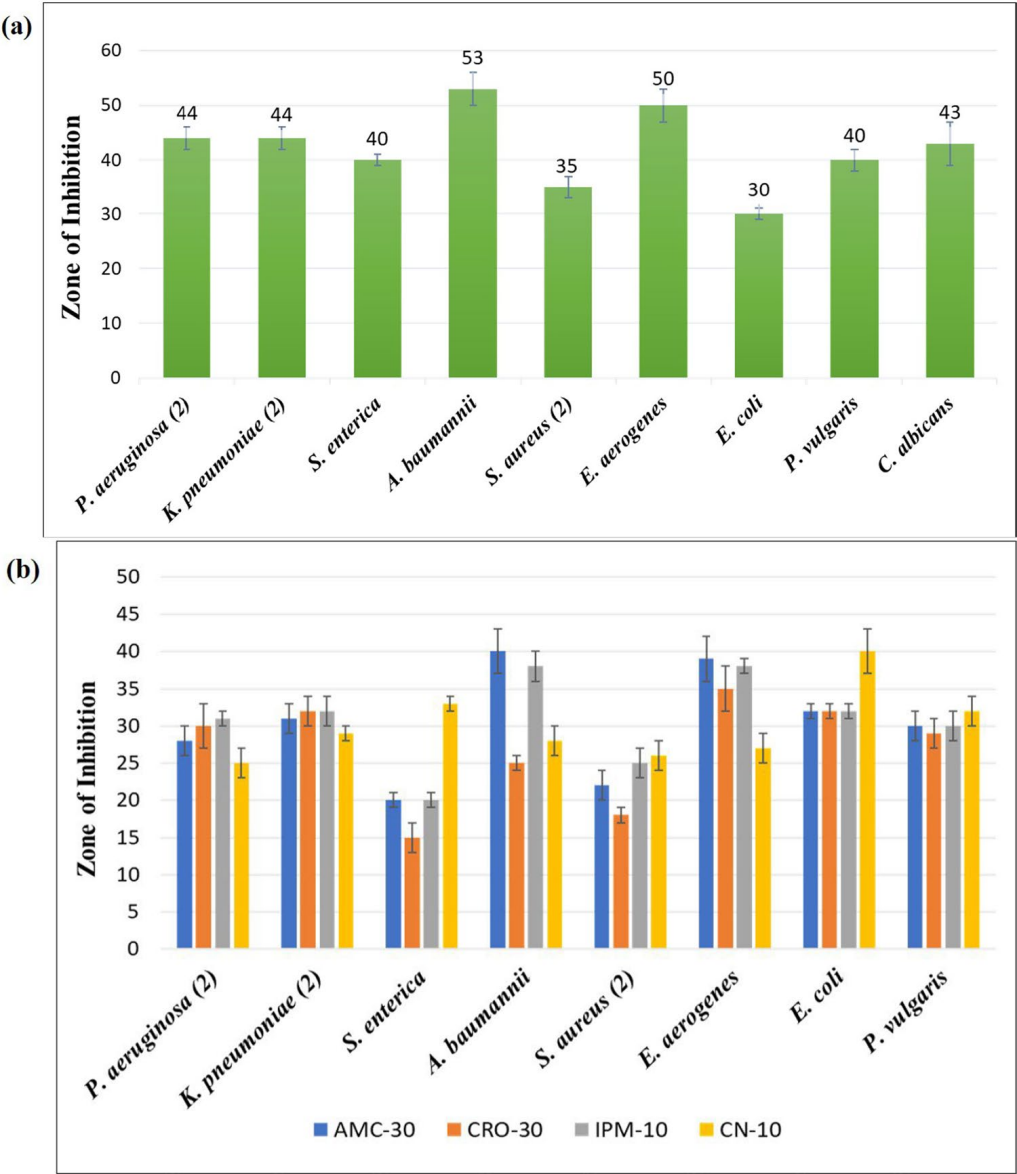
Graph summarizing zones of inhibition of **a** Cr_2_O_3_ nanoparticles against pathogenic microbial isolates, **b** combined antibacterial potential of Cr_2_O_3_ NPs and selected antibiotics

Crude fruit extract of *P. emblica* did not show any notable antimicrobial effect (Fig. 5a). On the other hand, *P. emblica*-mediated Cr_2_O_3_ nanoparticles showed excellent antimicrobial activity against both Gram-positive and Gram-negative bacterial isolates as well as the fungal isolate (Fig. 5c). Minimum zone of inhibition (30 mm) was recorded against *E. coli* and maximum zone of inhibition (53 mm) was seen against *A. baumannii*, which means that all the bacterial isolates were highly susceptible to the synthesized nanoparticles (Fig. 6a). Two isolates were included for each of *P. aeruginosa, K. pneumoniae* and *S. aureus*, and average zone diameters are described here. For all the other microbes, only one isolate was included. MIC values for Cr_2_O_3_ nanoparticles were observed as 0.2 µg/ml for *P. aeruginosa* and *E. aerogenes,* 0.6 µg/ml for *K. pneumoniae* and *S. aureus,* while 0.8 µg/ml for *S. enterica, A. baumannii, S. aureus, E. coli, P. vulgaris* and* C. albicans.*

### Antibiotic susceptibility testing

Various classes of antibiotics were used to test the susceptibility of bacterial isolates under study (Table 2, Fig. 5b). *S. aureus* and *E. coli* were sensitive to three out of eight antibiotics selected. *P. aeruginosa*, *A. baumannii* and *E. aerogenes* were sensitive to only one class of antibiotics tested while *K. pneumoniae*, *S. enterica* and *P. vulgaris* showed resistance to all antibiotics tested. Ciprofloxacin and ceftriaxone did not prove to be effective against any isolate. Colistin was effective against *P. aeruginosa* and *E. aerogenes*, while imipenem and gentamicin showed good inhibition of *S. aureus* and *E. coli.*

**Table 2:** Antibiogram of selected bacterial isolates

	Bacteria name	CL-10	IPM-10	CIP-5	CN-10	CRO-30	FEP-30	SCF-105	SXT-25
1	*P. aeruginosa*-1	S	R	R	R	R	R	R	–
2	*P. aeruginosa*-2	S	R	R	R	R	R	–	–
3	*K. pneumoniae*-1	–	S	R	R	R	–	R	–
4	*K. pneumoniae*-2	–	R	R	R	R	R	–	R
5	*S. enterica*	–	–	R	–	R	–	–	R
6	*A. baumannii*	–	R	R	R	R	R	S	R
7	*S. aureus*–1	–	S	–	S	R	–	–	S
8	*S. aureus*–2	–	S	–	R	S	–	–	S
9	*E. aerogenes*	S	R	R	R	R	R	R	
10	*E. coli*	–	S	R	S	R	S	–	R
11	*P. vulgaris*	–	R	R	R	R	R	–	R

### Synergistic activity of nanoparticles with antibiotics

All bacterial isolates under study were sensitive to Cr_2_O_3_ nanoparticles conjugated with the antibiotics (AMC-30, CRO-30, IPM-10 and CN-10) (Fig. 5d). Zones of inhibition were in the range of 15–40 mm (Fig. 6b). Results show that the antibacterial activity of Cr_2_O_3_ nanoparticles slightly decreased when they were used in combination with the antibiotics, which indicates a lack of synergism between the two. These results were also supported by the calculated fractional inhibitory concentration index (FICI) values which were greater than 1, meaning no synergism. Nanoparticles alone exhibited maximal antibacterial activity in case of all isolates, while antibiotics alone showed least effectiveness at all occasions.

### Anti-biofilm potential of nanoparticles

**Fig. 7:**
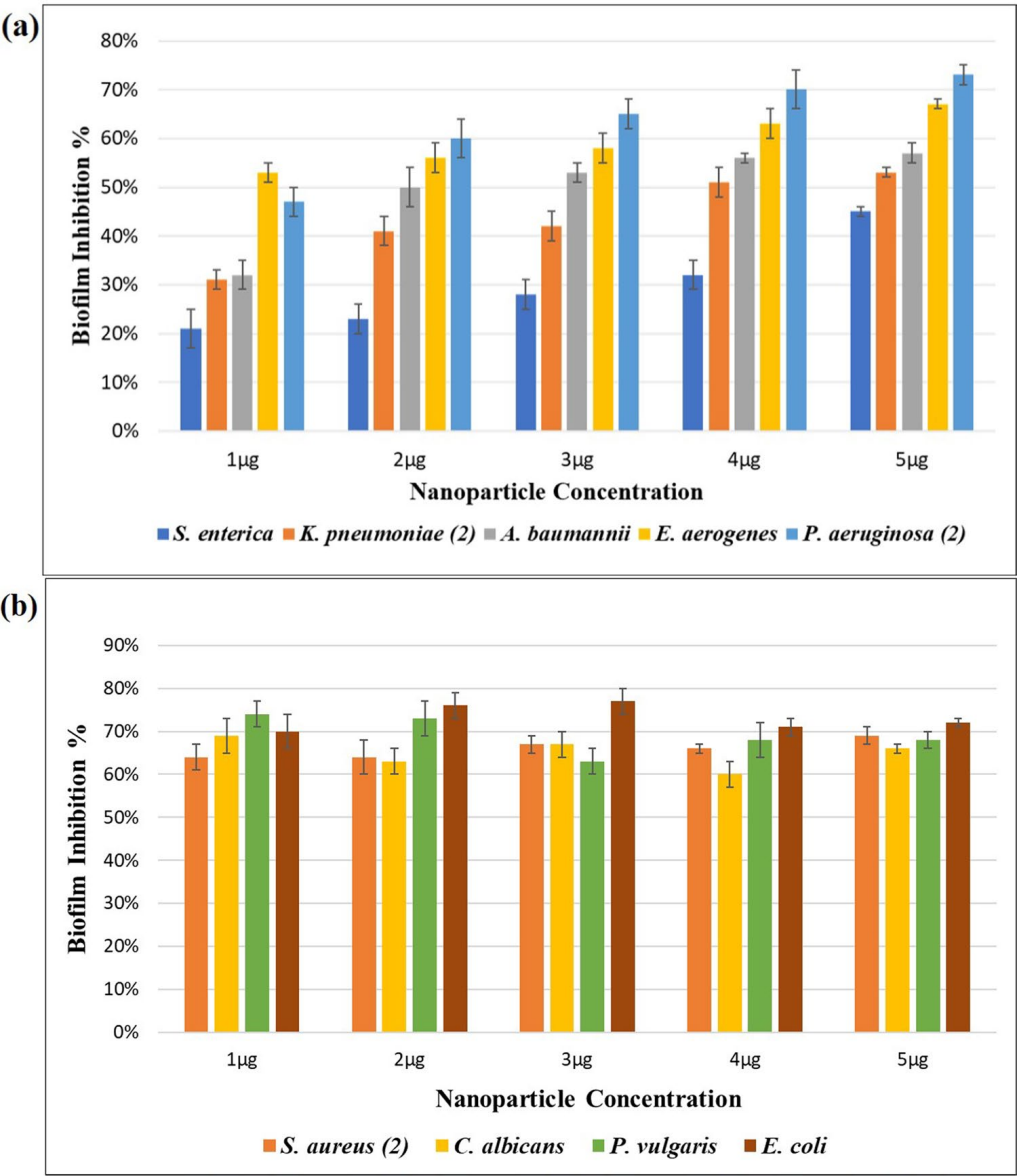
Graphs representing antibiofilm potential of Cr_2_O_3_ nanoparticles against various microbial isolates

Cr_2_O_3_ nanoparticles demonstrated good inhibition of biofilms produced by all the pathogenic microorganisms. A gradual decrease in absorbance values for wells was shown by the microtiter plate reader corresponding to the increasing nanoparticle concentration. The lowest nanoparticle test concentration was 1 µg/ml (0.001 mg/ml) which effectively showed 51% biofilm inhibition, on average. Highest test concentration 5 µg/ml (0.005 mg/ml) showed 63% biofilm inhibition on average. Least biofilm inhibition (45%) was noticed in case of *S. enterica* while maximum biofilm inhibition (77%) was observed in case of *E. coli.* Biofilm inhibition increased by increasing nanoparticle concentration in case of *P. aeruginosa, K. pneumoniae, S. enterica*, *A. baumannii* and *E. aerogenes* (Fig. 7a), while no such observation was recorded in case of *S. aureus, E. coli* and *P. vulgaris* (Fig. 7b)*.* Cr_2_O_3_ nanoparticles were also effective for inhibiting the biofilm formation by *C. albicans* up to 67%.

### Photocatalytic dye degradation potential

**Fig. 8:**
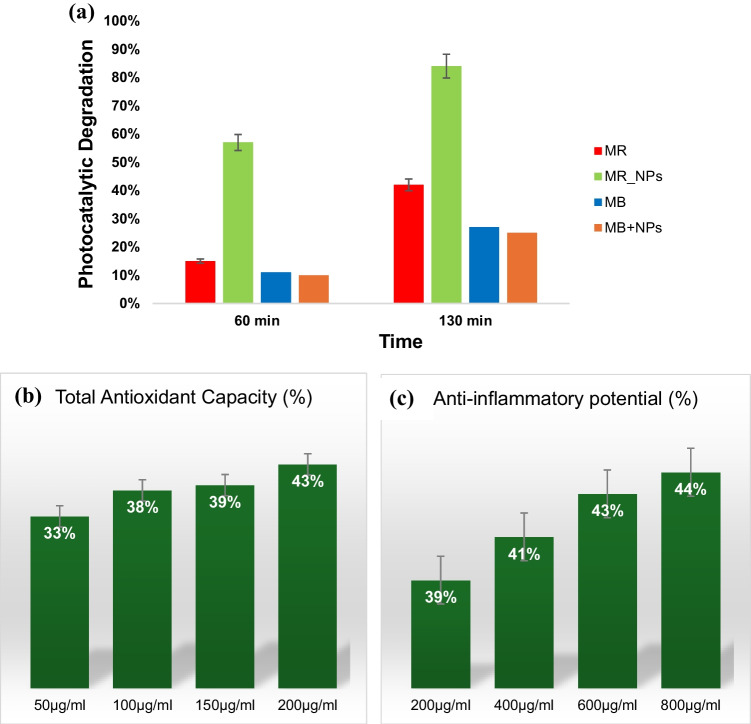
Graph representing **a** photocatalytic degradation of methyl red and methylene blue with and without Cr_2_O_3_ nanoparticles **b** total antioxidant capacity of Cr_2_O_3_ nanoparticles and, **c** anti-inflammatory potential of Cr_2_O_3_ nanoparticles

In the presence of sunlight, Cr_2_O_3_ nanoparticles successfully acted as catalyst for the degradation of methyl red dye, while no such observation was made in the case of methylene blue dye. When the reaction mixtures were exposed to sunlight for 60 min, methyl red degradation was 15% without nanoparticles and 57% with Cr_2_O_3_ nanoparticles (Fig. 8a). After 130 min, 42% of the total methyl red dye was degraded without nanoparticles, while 84% dye degradation was noticed along with Cr_2_O_3_ nanoparticles. Under same conditions, the final degradation of methylene blue dye (after 130 min) was 27% without nanoparticles, which remained unaffected upon the addition of Cr_2_O_3_ nanoparticles.

### Total antioxidant capacity and anti-inflammatory activity

Cr_2_O_3_ nanoparticles exhibited good antioxidant activity which increased with rising nanoparticles concentration. Most probably, the polyphenolic compounds present in *P. emblica* fruit extract (utilized in nanoparticles synthesis) acted as antioxidants. At concentrations of 50 µg/ml, 100 µg/ml, 150 µg/ml and 200 µg/ml, Cr_2_O_3_ nanoparticles showed 33%, 38%, 39% and 43% antioxidant activity, respectively (Fig. 8b). All tested concentrations of Cr_2_O_3_ nanoparticles also showed good anti-inflammatory potential which increased as the concentration of nanoparticles increased. At concentrations of 200 µg/ml, 400 µg/ml, 600 µg/ml and 800 µg/ml, Cr_2_O_3_ nanoparticles showed 39%, 41%, 43% and 44% anti-inflammatory potential, respectively (Fig. 8c). The total antioxidant capacity and anti-inflammatory activity, both were calculated by using ascorbic acid as a standard. The absorbance values calculated for Cr_2_O_3_ nanoparticles in case of both antioxidant and anti-inflammatory activities were lower than the absorbance values calculated for ascorbic acid at same concentrations and wavelengths.

## Discussion

Cr_2_O_3_ nanoparticles were efficiently prepared by utilizing *P. emblica* fruit extract as a reducing and stabilizing agent. The polyphenolic compounds and other phytochemicals present in *P. emblica* extract mainly acted as reducing agents in the biosynthesis of Cr_2_O_3_ nanoparticles. Phytochemical analysis confirmed the presence of such compounds. Caroling et al. also reported the presence of similar compounds in *P. emblica* fruit extract, including carbohydrates, tannis, saponins, flavonoids, alkaloids and phenols [18]. GC–MS analysis of the fruit extract of *P. emblica* revealed that the organic compound, bis (2-ethylhexyl) phthalate, was present in the highest proportions (81.48%). Endogenous phthalates have been consistently reported to exist in plants. A recent study reported advanced UPLC-MS analysis of the aqueous extract of *P. emblica* and reported a similar retention time (20.68 min) for ethyl-hexyl phthalate in *P. emblica* extract [24]. Bis (2-ethylhexyl) phthalate is among the most commonly used phthalates in various industries and is present in medical devices, electrical products, food packaging, building materials etc. Exposure to it can lead to adverse effects on health as it can act as an endocrine-disrupting chemical and is implicated in various pathological conditions among humans, particularly reproductive issues [25].

The formation of Cr_2_O_3_ nanoparticles was readily identified by a distinct color change to greenish black upon the addition of *P. emblica* fruit extract to chromium metal salt solution. A similar color change was observed by Hassan et al. upon the formation of α‐Cr_2_O_3_ nanoparticles using *Callistemon viminalis* flower extract [26]. Khan et al. synthesized Cr_2_O_3_ nanoparticles from *Abutilon indicum* leaf extract and reported black color of the final solution [27]. The color change happens due to the surface plasmon resonance (SPR) phenomenon which occurs when free electrons on the surface of metallic nanoparticles oscillate in resonance with incident light, resulting in selective light absorption and scattering that gives rise to characteristic colors. The slight variation in color possibly stems from the type of green source, metal salt and the synthesis method used. Although the above studies successfully demonstrated the formation of Cr_2_O_3_ nanoparticles using different natural reducing agents, the mechanism underlying the reduction and stabilization of nanoparticles using plant extracts is not yet fully understood and requires further investigation.

The results of UV–visible spectroscopy showed peaks between 300 and 500 nm. Sharp peaks appeared at 350 nm and 450 nm. The peak at 450 nm signifies the d-d transition of the metal during nanoparticle synthesis. Sharma and Sharma as well as Zainab et al. reported similar UV–visible spectra regarding Cr_2_O_3_ nanoparticles synthesized via green route and observed single peaks at 460 nm and 425 nm, respectively, but Iqbal et al. observed two peaks at 269 nm and 369 nm [28–30]. Although, sharp peaks are documented at slightly different wavelengths but, in general, SPR for Cr_2_O_3_ nanoparticles lies in the range of 250–450 nm [31]. For potassium dichromate solution, two peaks appeared at 257 nm and 350 nm, which were similar to those reported by Burke and Mavrodineanu [32]. UV–visible spectrum of *P. emblica* fruit extract revealed a single sharp peak at 310 nm. Saif et al. also documented the absorption peak of *P. emblica* leaf extract in the region of 300–350 nm, which is quite similar to the results reported in the present study [33].

FTIR spectrum of Cr_2_O_3_ nanoparticles showed peaks at 3779 cm^−1^, 3297 cm^−1^, 2308 cm^−1^, 1516 cm^−1^ and 1390 cm^−1^ which pointed towards various functional groups, while the FTIR signals at 946 cm^−1^, 759 cm^−1^ and 641 cm^−1^ indicated Cr–O bond which validated the formation of Cr_2_O_3_ nanoparticles. Iqbal et al. reported a similar FTIR spectrum regarding green synthesized Cr_2_O_3_ nanoparticles showing peaks at 3396 cm^−1^, 972 cm^−1^, 609 cm^−1^ and 527 cm^−1^ [28]. Ahmed Mohamed et al. used *Hyphaene thebaica* fruit extracts to demonstrate the biosynthesis of Cr_2_O_3_ nanoparticles. The FTIR spectrum documented by them showed peaks at 3405 cm^−1^, 3060 cm^−1^, 1630 cm^−1^, 949 cm^−1^, 647 cm^−1^, 565 cm^−1^ and 417 cm^−1^ which were similar to the ones reported in the present study [34]. However, a slight difference in the number and position of peaks could be observed due to the varying nature of sample (solid or liquid) and the green source used for nanoparticle synthesis. FTIR spectroscopy of *P. emblica* extract revealed peaks at 3342 cm^−1^ and 1634 cm^−1^ signifying the presence of phenols and alcohols. Similar results were reported by a previous study, as well [35]. FTIR of potassium dichromate revealed peaks at 950 cm^−1^ and 748 cm^−1^ indicating metal oxide bonds which was in accordance with the previous literature [36]. Many more peaks were observed in the FTIR spectrum of nanoparticles as compared to that of fruit extract. One plausible explanation for this could be the presence of various biomolecules in the fruit extract such as proteins, polysaccharides, flavonoids, phenolics etc., which act as capping agents during the synthesis of nanoparticles by adsorbing onto their surface. These phytochemicals also undergo chemical transformation by acting as reducing and chelating agents during nanoparticle formation. Together, these processes can introduce new functional groups and vibrational modes leading to the observation of additional peaks in FTIR spectrum [37].

XRD analysis of Cr_2_O_3_ nanoparticles revealed the presence of multiple peaks which were compared with the standard XRD patterns given by the Joint Committee on Powder Diffraction Standards (JCPDS). A study done by Tsegay et al. showed the synthesis of Cr_2_O_3_ nanoparticles using cactus plant extract and reported an XRD pattern exhibiting intensity peaks at 24.6°, 33.7°, 36.1°, 39.4°, 41.3°, 44.1°, 50.2°, 54.8°, 58.6°, 63.2°, 65.2°, 73.5°, 76.9°, and 79.2° diffraction angles that corresponded to reflections from (0 1 2), (1 0 4), (1 1 0), (0 0 6), (1 1 3), (2 0 2), (0 2 4), (1 1 6), (1 2 2), (2 1 4), (3 0 0), (1 1 9), (2 2 0), and (3 0 6) reticular planes, respectively [38]. In another study, Rakesh et al. used electrochemical method to synthesize Cr_2_O_3_ nanoparticles with *Mukia maderaspatana* plant extract. XRD spectrum documented by them showed clearly distinguishable peaks for crystalline Cr_2_O_3_ having 2θ values of 24.6°, 36.3°, 50.2° and 63.62°, which corresponded to the crystal planes of (0 1 2), (1 1 0), (0 2 4) and (2 1 4), respectively [39]. The results of both these studies are quite similar to the ones reported in the present study in terms of peak positions and crystal planes.

EDX graph showed Cr and O peaks which were located between 0.0 and 0.6 keV indicating the presence of chromium and oxygen elements in the sample. Potassium was also present in notable amounts in the nanoparticle sample. These results showed a high degree of similarity to those reported by Satgurunathan et al. in case of *Allium sativum*-mediated Cr_2_O_3_ nanoparticles, presenting Cr and O peaks in the same positions as the ones reported in this study [40]. Iqbal et al. also reported a similar EDX spectrum regarding green synthesized Cr_2_O_3_ nanoparticles [28]. SEM revealed agglomeration of nanoparticles which resulted in the formation of large flakes which were polymorphic in terms of shape. Previously, silver nanoparticles synthesized from seed extract of *Coffea arabica* were reported to possess polymorphic shapes such as ellipsoidal, rocky, spherical, flake type as well as irregular agglomerates [41]. In another study, nanoparticles were synthesized using *Planomicrobium* sp. and their antibacterial activity was evaluated against foodborne pathogens. They also reported a lot of agglomeration of particles forming irregular shapes [42]. Such morphology can arise owing to the presence of biomolecules on the surface of nanoparticles. These biomolecules are responsible for capping and stabilization of the nanoparticles during synthesis [43].

*Phyllanthus emblica*-mediated Cr_2_O_3_ nanoparticles revealed extraordinary antimicrobial activity against bacteria and fungi. Minimum zone of inhibition (30 mm) was recorded against *E. coli*, and maximum zone of inhibition (53 mm) was seen against *A. baumannii*, which means that all the bacterial isolates were highly sensitive to these nanoparticles. 43 mm zone of inhibition was noticed against *Candida albicans*. A previous study dealt with the synthesis of Cr_2_O_3_ nanoparticles using *Abutilon indicum* leaf extract and reported maximum zone of inhibition around 20 mm, against *S. aureus* [28]. Another study regarding silver nanoparticles synthesized using fruit extract of *Phyllanthus emblica* documented good antimicrobial activity against *S. aureus* and *K. pneumoniae* [21]. *P. emblica*-mediated Cr_2_O_3_ nanoparticles have clearly shown superior antimicrobial activity as compared to the previously synthesized nanoparticles using different sources.

In the present study, 0.6 µg/ml concentration was observed as an average MIC value for Cr_2_O_3_ nanoparticles. The least observed value was 0.2 µg/ml, while the highest MIC score was 0.8 µg/ml. These values were lower than the previously reported MIC values. Iqbal et al. reported MIC of green synthesized Cr_2_O_3_ nanoparticles in the range of 5 μg/ml to 100 μg/ml against a diversity of bacterial isolates [28]. Loo et al. synthesized silver nanoparticles using pu-erh tea leaf extract and proclaimed their MIC value as 3.9–7.8 μg/ml against various pathogenic bacteria [44]. The difference between the reported values may be because of varying physio-chemical properties of nanoparticles, nature of microorganisms and most importantly, the concentration of nanoparticles used. Antibiotic susceptibility testing of the selected bacterial isolates revealed that they were resistant to multiple drugs and could be termed as multi-drug resistant (MDR) bacteria. Wang et al. also reported the prevalence of such MDR bacteria in patients having nosocomial infections, which showed resistance to ampicillin, ceftriaxone, ciprofloxacin and imipenem, while sensitivity to amikacin and gentamicin [45]. These results showed a high degree of similarity to the results reported in the present study.

The incorporation of metallic nanoparticles with antimicrobial agents presents a highly promising strategy to address the challenge of microbial antibiotic resistance. This pioneering method entails merging metal oxide nanoparticles with antimicrobial agents, leading to the creation of a new class of antimicrobial medications that demonstrate synergistic effects against drug-resistant bacteria [46]. *P. emblica*-mediated Cr_2_O_3_ nanoparticles did not reveal synergism with any of the selected antibiotics, as revealed by the calculated FICI values. Contrarily, Nishanthi et al. reported good synergism between gold, silver, and platinum nanoparticles when conjugated with different classes of antibiotics. Gold nanoparticles exhibited 100% increase in the antibacterial activity, whereas silver and platinum nanoparticles showed 87.5% and 37.5% increment, respectively [47]. The synergistic effect may be due to the bond formation between antibiotics and nanoparticles.

*P. emblica*-mediated Cr_2_O_3_ nanoparticles demonstrated good biofilm inhibition against all the pathogenic microorganisms at all concentrations tested. Younis et al. used *Rosa floribunda charisma* extract to synthesize magnesium nanoparticles and reported 80% biofilm inhibition against *S. epidermidis* and *P. aeruginosa* at concentrations of 1–2 µg/ml and 4 µg/ml, respectively [48]. In the present study, 70% biofilm inhibition was observed against *P. aeruginosa* at a similar concentration. The similarity in results manifests the competency of green synthesized nanoparticles in biofilm inhibition. In the present study, an improved degradation of methyl red dye was observed upon addition of Cr_2_O_3_ nanoparticles, while the degradation of methylene blue dye remained unaffected. A previous study documented the photocatalytic degradation of methyl red and methylene blue dye by multi-functional CuO nanoparticles as 85% and 90%, respectively [49]. Providing same conditions and time period, 84% photocatalytic degradation of methyl red was noticed in the current study, while methylene blue degradation was 26%, which remains unexplained. As the nature of interaction between nanoparticles and dye molecules defines the extent of dye degradation, a difference in results may appear with varying types of nanoparticles and dyes.

The determination of the total antioxidant capacity of Cr_2_O_3_ nanoparticles was carried out using the phosphomolybdenum method. The results indicated that nanoparticles at various concentrations displayed significant antioxidant capacity, suggesting the presence of modulators that effectively combat superoxide radicals. At 200 µg/ml concentration, Cr_2_O_3_ nanoparticles showed 33% antioxidant capacity. Similar results were reported by Nivethitha and Rachel, who measured 35% anti-oxidant activity of honey-mediated Cr_2_O_3_ nanoparticles at the same concentration [50]. Cr_2_O_3_ nanoparticles showed good anti-inflammatory activity which increased with increasing nanoparticle concentration. At 200 µg/ml, Cr_2_O_3_ nanoparticles showed 39% anti-inflammatory potential. Aafreen et al. used ginger oil to synthesize silver nanoparticles and reported maximum anti-inflammatory activity showing 90% inhibition at 60 µg/ml concentration [51]. Variation in results could be noticed because of the usage of different plant sources and techniques for nanoparticle production, which ultimately affects the composition and interactions of nanoparticles with other molecules.

Our research focused on investigation of in vitro biological activities of the synthesized nanoparticles but did not examine their mode of action. Studying the underlying mechanism of action could provide a better understanding about the nature of nanoparticles and their possible applications. Optimization of protocol regarding nanoparticle synthesis is also needed to produce stable nanoparticles having a narrow size distribution. Moreover, biosafety and biocompatibility studies should be a prime focus in future research, which could confirm the purity and non-toxic nature of the nanoparticles.

## Conclusion

This study provides a clear, reproducible and eco-friendly approach to synthesize Cr_2_O_3_ nanoparticles using *P. emblica* fruit extract as a reducing and stabilizing agent. These nanoparticles were characterized by using techniques like FTIR spectroscopy, UV–visible spectroscopy, XRD, EDX and SEM. The extraordinary antibacterial and antifungal activities shown by the nanoparticles against various clinical isolates exceeded the antibacterial activity of various antibiotics. Nanoparticles also effectively inhibited biofilm formation by isolated microorganisms. *P. emblica*-mediated Cr_2_O_3_ nanoparticles showed good antioxidant and anti-inflammatory activities, as well. Photocatalytic degradation of methyl red dye was also accelerated by these nanoparticles. These results suggest that green-synthesized Cr_2_O_3_ nanoparticles hold the potential to be incorporated in medical field as effective antibacterial and antifungal agents. Moreover, they are well-suited for environmental remediation purposes like dye degradation. These nanoparticles can become prominent therapeutic agents in future, if explored further.

## Experimental

### Synthesis of P. emblica fruit extract

Fruits were separated from the plants of *P. emblica* which were purchased from the local nursery in Multan, Pakistan. Fruits were washed, air-dried and grinded to obtain a fine powder. Aqueous extract was prepared in three different concentrations i.e., 2%, 4%, and 8%. Solutions were heated for half an hour at 80 °C with constant stirring. Prepared extract was cooled down, filtered and instantly used to synthesize Cr_2_O_3_ nanoparticles.

### Phytochemical and GC–MS analysis of aqueous fruit extract

For phytochemical testing, freshly prepared and filtered extract of dried *P. emblica* fruit powder was utilized. According to the established protocols, tests were run to check for the presence of carbohydrates, flavonoids, alkaloids, steroids, terpenoids, glycosides, tannins and phenols. Gas chromatography–mass spectroscopic (GC–MS) analysis of fruit extract of *Phyllanthus emblica* was performed using the equipment GCMS-5977B Agilent Technologies USA. A DB-1 standard column with dimensions of 25 m × 0.250 mm × 0.25 µm was utilized for this purpose, and helium was used as a carrier gas with a flow rate of 1 ml/min. The equipment’s injector was operated at a temperature of 250 °C and pressure of 8.8085psi.

### ***P. emblica-mediated synthesis of Cr***_***2***_***O***_***3***_*** nanoparticles***

**Fig. 9:**
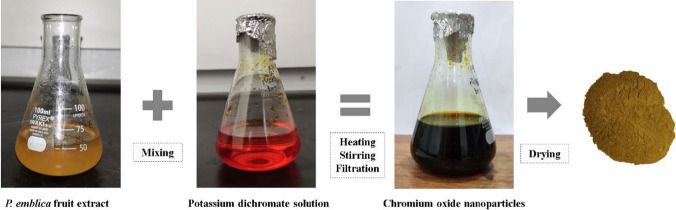
A schematic diagram showing the synthesis of Cr_2_O_3_ nanoparticles from *P. emblica* fruit extract

For the preparation of Cr_2_O_3_ nanoparticles, 10% potassium dichromate solution was prepared in water and stirred for half an hour to get a uniform bright orange solution. 15–20 ml of freshly prepared *P. emblica* fruit extract was added gradually to the potassium dichromate solution. An instant dark green-black color was noticed, which confirmed the formation of Cr_2_O_3_ nanoparticles. The solution was then stirred and heated at 80 °C for 30 min, cooled down to room temperature and filtered. Nanoparticles were dried in hot air oven and crystals were grinded to get a fine powder (Fig. 9). Cr_2_O_3_ nanoparticles were prepared with varying concentrations of *P. emblica* fruit extract by using the same procedure and the concentration with the best bacterial inhibition was used for further tests.

### ***Characterization of Cr***_***2***_***O***_***3***_*** nanoparticles***

Various techniques were used to determine the physical and chemical characteristics of synthesized nanoparticles. To initially confirm the formation of Cr_2_O_3_ nanoparticles, UV–visible measurements were conducted within the range of 200 and 600 nm. Moreover, the chemical properties of nanoparticles were observed by Fourier transform infrared spectroscopy (FTIR). FTIR spectra were measured within the range of 500–4000 cm^−1^. The crystalline nature of Cr_2_O_3_ nanoparticles was explored through X-ray diffraction (XRD) analysis using the standard X-ray beam ranging between 50 and 500 µm (X-Ray Diffractometer BRUKER D8 Discover). Scanning electron microscopy (SEM) was employed to examine the size and shape as well as the mechanical properties of Cr_2_O_3_ nanoparticles whereas, energy dispersive X-ray analysis (EDX) was used to determine the elemental composition of the nanoparticles. For this purpose, the solution containing nanoparticles was lyophilized and converted to a fine powder. SEM–EDX analysis (EDAX Team—IT100LA) was then performed at a voltage of 20.0 kV, a current of 3.97 A, and a magnification up to 1000x.

### ***Antimicrobial activity of Cr***_***2***_***O***_***3***_*** nanoparticles***

The agar well-diffusion method was used to investigate the antimicrobial activity of Cr_2_O_3_ nanoparticles against clinical pathogens. A total of 11 bacterial isolates (*Pseudomonas aeruginosa, Staphylococcus aureus, Klebsiella pneumoniae, Proteus vulgaris, Escherichia coli, Enterobacter aerogenes, Salmonella enterica*, *Acinetobacter baumannii*) and 1 fungal isolate (*Candida albicans*) were selected for this purpose. All the test microbes were standardized using 0.5 McFarland standard and swabbed onto the Mueller–Hinton agar (MHA) plates. After drying, three wells were made on each petri plate at an appropriate distance. 50 µl nanoparticle solution was filled in each well and plates were kept in the incubator at 37 °C overnight. After incubation, zones of inhibition were measured in accordance with the CLSI guidelines, and results were recorded.

### Determination of MIC

MIC of Cr_2_O_3_ nanoparticles was determined by using the standard broth microdilution method. All the test microorganisms were grown overnight in nutrient broth and standardized. A sterile 96-well plate was taken, and its wells were filled with 100 µl nutrient broth. Two-fold dilutions of nanoparticles were made and 50 µl of standardized microbial suspension was added in each of the first 10 wells. Positive and negative controls were set in the next two wells. The plates were incubated at 37 °C for 24 h. MIC was determined by recording absorbance values at a wavelength of 620 nm using a microtiter plate reader.

### Antibiotic susceptibility testing of microbial isolates

Antibiotic susceptibility profiling of bacterial isolates was done by Kirby-Bauer disc diffusion method. MHA plates were prepared, and bacterial isolates were inoculated. Different antibiotic discs including Clindamycin (CL-10), Imipenem (IPM-10), Ciprofloxacin (CIP-5), Gentamicin (CN-10), Ceftriaxone (CRO-30), Cefepime (FEP-30), Sulbactam (SCF-105) and Trimethoprim/Sulfamethoxazole (SXT-25) were placed on each plate at a suitable distance. Prepared plates were placed in the incubator at 37 °C for 24 h. After incubation, zones of inhibition were measured, and the results were recorded according to the CLSI guidelines.

### ***Synergistic activity of Cr***_***2***_***O***_***3***_*** nanoparticles with antibiotics***

The synergistic effect of Cr_2_O_3_ nanoparticles was checked with Ceftriaxone (CRO-30), Augmentin (AMC-30), Imipenem (IPM-10) and Gentamicin (CN-10). These antibiotics were selected on the basis of antibiotic sensitivity test results: most of the bacterial isolates were resistant to these antibiotics while only a few were sensitive. MHA plates were prepared, and bacterial isolates were swabbed onto them. Then, antibiotic discs soaked in nanoparticle solution were aseptically placed on the plates which were then incubated at 37 °C overnight. Subsequently, zones of inhibition were measured, and the results were recorded. To verify synergism, FICI values were determined by using the formula given below:

$$FICI = \frac{Antibacterial \,effect \,of \,NPs+Antibacterial \,effect \,of \,antbiotic}{Antibacterial \,effect \,of \,both \,NPs \,\& \,antibiotic}$$

### ***Antibiofilm potential of Cr***_***2***_***O***_***3***_*** nanoparticles***

To determine the anti-biofilm potential of Cr_2_O_3_ nanoparticles, the protocol described by Mohanta et al. was followed with some modifications [52]. A sterile 96-well microtiter plate containing Mueller–Hinton broth (MHB) was used for this purpose. Bacterial isolates were grown overnight in MHB, standardized and added to the wells. Negative and positive controls were included. Various dilutions of Cr_2_O_3_ nanoparticles were then added to the test wells and plates were incubated at 37 °C for 48 h. Following the incubation period, wells were emptied, washed gently with phosphate buffered saline (PBS) and air-dried. Sodium acetate (2% w/v) was used to fix the adherent bacteria after which the wells were flooded with crystal violet (0.1% w/v) stain and plates were kept in dark for half an hour. Sterile distilled water was used to wash wells in order to remove the excessive dye. Plates were air-dried and 95% ethanol was filled in each well. Microtiter plate reader was used to obtain absorbance at 620 nm. The following formula was used to find out the percentage biofilm inhibition:

$$\% {\text{ biofilm inhibition }} = \, [{1} - ({\text{OD}}_{620} {\text{of wells treated with NPs}}/{\text{OD}}_{620} {\text{of the non}} - {\text{treated control}}) \, \times { 1}00]$$

### ***Photocatalytic dye degradation by Cr***_***2***_***O***_***3***_*** nanoparticles***

Photocatalytic degradation of methyl red and methylene blue dyes was observed in the presence of Cr_2_O_3_ nanoparticles, by following the protocol documented by Dulta et al. [47]. Dye solutions (20 ppm) were prepared in separate beakers and initial dye concentration was measured. 10 mg/L of Cr_2_O_3_ nanoparticles was added to both dye solutions, stirred for 15 min in the dark and exposed to the sunlight for 130 min. At the time of experiment, temperature was 32–35 °C. During sunlight exposure, the solutions were stirred from time to time. After reaction time, nanoparticles were removed from the solution by centrifugation and the concentration of methyl red and methylene blue dyes was measured by using UV–visible spectrophotometer at 525 nm and 625 nm, respectively. The dye degradation percentage was measured as:

$$\upeta = \frac{{\text{C}}0-{\text{Ct}}}{C0} \times 100$$

where, C_0_ is the initial dye concentration and Ct is the final dye concentration after a certain exposure time.

### ***Antioxidant potential of Cr***_***2***_***O***_***3***_*** nanoparticles***

Total antioxidant capacity (TAC) of Cr_2_O_3_ nanoparticles was determined by using phosphomolybdenum method. 4 mM ammonium molybdate, 28 mM sodium phosphate and 0.6 M sulfuric acid were mixed in equal amounts to prepare the working reagent. Various concentrations of nanoparticles ranging from 50 to 200 µg/ml were prepared in distilled water. Reagent solution was mixed with different nanoparticle dilutions in test tubes. Ascorbic acid was used as a control. Test tubes were incubated in a water bath at 95 °C for 90 min. In the presence of antioxidants, greenish-blue color appears indicating the reduction of phosphomolybdate ion and the creation of phosphomolybdenum (V) complex. After incubation, absorbance of each sample was recorded at 695 nm. Percentage TAC was calculated by using the formula given below.

$${\text{TAC }}\% \, = \, ({\text{OD}}_{{{\text{control}}}} - {\text{OD}}_{{{\text{sample}}}} ) \, /{\text{ OD}}_{{{\text{control}}}} \times {1}00$$

The experiment was conducted in triplicates and the mean value was calculated.

### ***Anti-inflammatory activity of Cr***_***2***_***O***_***3***_*** nanoparticles***

For the evaluation of anti-inflammatory effect of Cr_2_O_3_ nanoparticles, a working solution of 0.2% Bovine serum albumin (BSA) was prepared. Various concentrations of nanoparticles ranging from 200-800 µg/ml were prepared and BSA was added to those separately in the test tubes. Test tubes were incubated in the water bath at 75 °C for 5 min and then cooled down. Ascorbic acid served as the control. The absorbance of each sample was recorded at 660 nm, and the percentage anti-inflammatory activity was calculated using the formula:

$${\text{Anti - inflammatory activity}}\,\% = ({\text{OD}}_{{{\text{control}}}} - {\text{OD}}_{{{\text{sample}}}} ) \, /{\text{ OD}}_{{{\text{control}}}} \times { 1}00$$

The experiment was conducted in triplicates and the mean value was calculated.

## Data Availability

All data generated or analyzed during this study are available from the corresponding author on reasonable request.

## Declarations

### Plant guidelines

We followed the local guidelines for obtaining fruit of *Phyllanthus emblica*, also known as Indian gooseberry. The plant was obtained from a collection of cultivated plants in a local nursery. No permissions or licenses were required for this purpose.

### Permission to collect the plants

No relevant permits/permissions/licenses were required to collect plants from local nursery.
